# Altering an extended phenotype reduces intraspecific male aggression and can maintain diversity in cichlid fish

**DOI:** 10.7717/peerj.209

**Published:** 2013-11-26

**Authors:** Isabel Santos Magalhaes, Guy E. Croft, Domino A. Joyce

**Affiliations:** 1 School of Biological, Biomedical and Environmental Sciences, University of Hull, Hull, UK; 2 School of Life Sciences, University of Nottingham, Nottingham, UK; 3 Petersfield House, Dog Kennel Lane, Hadlow Down, East Sussex, UK

**Keywords:** Male aggression, Extended phenotype, Negative frequency-dependent intrasexual selection, Female preference, Haplochromine cichlid, Lake Malawi, Bower

## Abstract

Reduced male aggression towards different phenotypes generating negative frequency-dependent intrasexual selection has been suggested as a mechanism to facilitate the invasion and maintenance of novel phenotypes in a population. To date, the best empirical evidence for the phenomenon has been provided by laboratory studies on cichlid fish with different colour polymorphisms. Here we experimentally tested the hypothesis in a natural population of Lake Malawi cichlid fish, in which males build sand-castles (bowers) to attract females during seasonal leks. We predicted that if bower shape plays an important role in male aggressive interactions, aggression among conspecific males should decrease when their bower shape is altered. Accordingly, we allocated randomly chosen bowers in a *Nyassachromis* cf. *microcephalus* lek into three treatments: control, manipulated to a different shape, and simulated manipulation. We then measured male behaviours and bower shape before and after these treatments. We found that once bower shape was altered, males were involved in significantly fewer aggressive interactions with conspecific males than before manipulation. Mating success was not affected. Our results support the idea that an extended phenotype, such as bower shape, can be important in maintaining polymorphic populations. Specifically, reduced male conspecific aggression towards males with different extended phenotypes (here, bower shapes) may cause negative frequency-dependent selection, allowing the invasion and establishment of a new phenotype (bower builder). This could help our understanding of mechanisms of diversification within populations, and in particular, the overall diversification of bower shapes within Lake Malawi cichlids.

## Introduction

The mechanisms which lead to species divergence in secondary sexual characters are relatively poorly understood and their potential to lead to speciation has remained contentious (Arnegard & Kondrashov, 2004; Coyne & Orr, 2004). This is because evolutionary change through sexual selection can only work if there is enough standing genetic variation to produce both the necessary divergence in secondary sexual traits, and the corresponding mating preference for those traits. This would allow the stabilization of trait polymorphisms and eventual population divergence (Arnegard & Kondrashov, 2004).

Negative frequency-dependent selection has been suggested as a mechanism by which sexual selection can maintain polymorphisms in populations, and eventually lead to speciation (Mikami, Kohda & Kawata, 2004; Seehausen & Schluter, 2004; Van Doorn, Dieckmann & Weissing, 2004). In systems where the same trait is under selection by males in aggressive encounters, and by females in mate choice, selection can confer rare phenotypes an advantage. If individuals direct more aggression towards rivals that resemble their own phenotype than towards alternative phenotypes (Pauers et al., 2008), the rare phenotypic forms will enjoy an advantage through reduced aggression. This would allow the invasion of a new phenotype into a population, which could then increase in number. Eventually, as the new phenotype becomes more common, its relative advantage would begin to decrease due to an increased rate of incurred attacks. The frequency of the new phenotype is then expected to oscillate, with negative frequency-dependent selection generated by intrasexual aggression allowing the emergence of a balanced polymorphism (Mikami, Kohda & Kawata, 2004; Seehausen & Schluter, 2004; Van Doorn, Dieckmann & Weissing, 2004). Eventually, if alternative female preference becomes linked to the male trait, this could allow the possibility of reproductive isolation and speciation (Fisher, 1930). Initial divergence in secondary sexual characters which lead to biases in territorial aggression may also enhance divergence through agonistic character displacement (Grether et al., 2009). By altering the traits involved in competitor recognition, the trait owner may escape the effect of interference competition. These processes have been recently reviewed in Qvarnström, Vallin & Rudh (2012) and Dijkstra & Groothuis (2011).

The haplochromine cichlid fish from the African Rift Valley Lakes are known as some of the most spectacular adaptive radiations (Kocher, 2004). In particular Lakes Victoria and Malawi each contain hundreds of species which have emerged relatively rapidly (Genner et al., 2007). These fish are also well known for their divergence in secondary sexual characters (Allender et al., 2003; Kocher, 2004). Laboratory studies on cichlid fish from Lake Victoria have supported the hypothesis that rare phenotypes may have an advantage by showing that both males (Dijkstra et al., 2006) and females (Dijkstra, Seehausen & Groothuis, 2008) attacked individuals of colour similar to their own more often than those of different colour. However, negative frequency-dependent sexual selection via reduced aggression has not yet been demonstrated in the natural environment. Here, we use a sand-dwelling species of a haplochromine cichlid from Lake Malawi to test whether individuals with a rare extended phenotype receive less aggression in the wild.

The “sand-dwelling” lineage of bower-building cichlid fish from Lake Malawi represents an estimated 200 species, approximately 25% of the diversity of Lake Malawi cichlid fish (Konings, 2007). Relatively little is known about them, but an increasing number of studies have focused on their fascinating behaviour (Genner et al., 2008; Young et al., 2009; Martin & Genner, 2009; Young et al., 2010; Martin, 2010). These fish typically congregate in leks once or twice a year (McKaye, 1983 and personal observation, DJ), where they build and defend species-specifically shaped sand mounds (i.e., bowers) on which they mate with females. These structures are analogous to the decorative “bowers” of bird studies (Diamond, 1986; Borgia, 1995) since they are constructed with the single purpose of attracting females and once mating has occurred, the female leaves with the fertilized eggs in her mouth. Several different species can coexist and build bowers in the same location and bower shapes are species specific (Kidd, Kidd & Kocher, 2006) but little is known about the forces which produce and maintain the diversity in bower shape in multi-species leks. Bower shape is selected by females (Mckaye, Louda & Stauffer, 1990; Taylor et al., 1998; Kellogg, Stauffer & Mckaye, 2000; Young et al., 2010), but recent work has demonstrated that males also use them as an indication of social status (Genner et al., 2008; Martin & Genner, 2009). Thus, these secondary sexual traits which are malleable extended phenotypes provide a unique opportunity to examine the role of divergence of sexually selected traits in population separation and speciation (Stauffer, LoVullo & McKaye, 1993; Schaedelin & Taborsky, 2009).

Here, we tested the possibility that conspecific intrasexual aggression is directed away from divergent phenotypes which could allow diversification of bower phenotypes within leks. We studied a lek of the bower-building cichlid *Nyassachromis* cf. *microcephalus* (Konings, 2009, pers. comm.) at Thumbi West Island, in southern Lake Malawi (Video S1). This lek is maintained for several weeks between July and September (personal observation, DJ). We manipulated bowers to a different shape and measured mating and aggression behaviours before and after the changes in bower shape were made. We predicted that males would be involved in fewer aggressive interactions after their bower was changed to a different shape. If males with the different bower shape experience a negatively frequency-dependent advantage through reduced aggression from other males, then this could allow for the establishment of a balanced polymorphism even if female preference is not (yet) linked to bower shape. In such a case, if female mate preference eventually becomes linked to bower shape, populations with divergent bower shape could become reproductively isolated.

## Material and Methods

### Study system and sampling site

**Figure 1:**
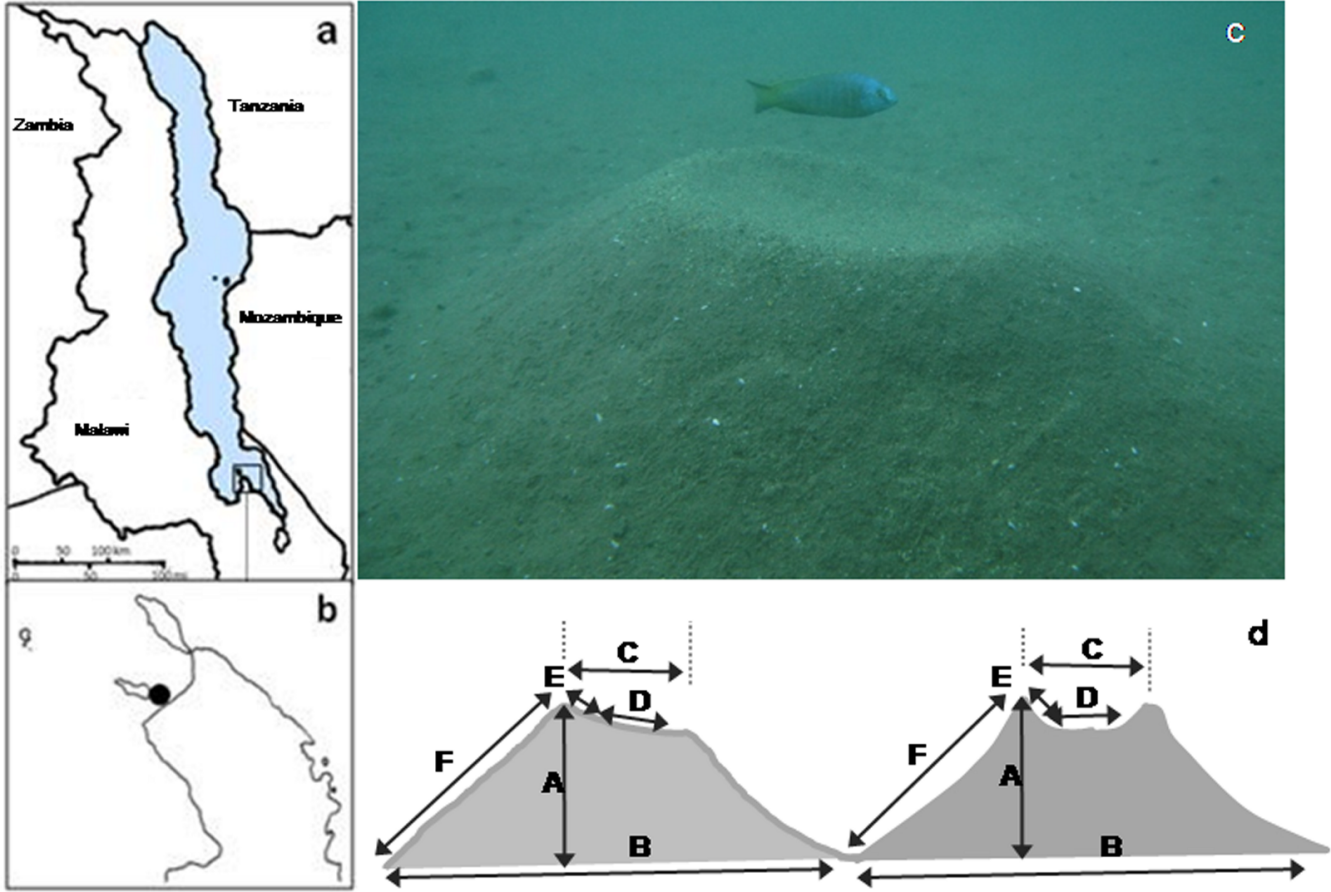
Study sites, lek location, study species and bower shapes and measurements. (A) Map of Lake Malawi showing (B) the southern arm of the lake and the location of the lek; (C) *Nyassachromis* cf. *microcephalus* male over his bower, and (D) left hand side original bower shape with standard measurements taken (A–F) and right hand side, bower shape after manipulation.

Our study species, *Nyassachromis* cf. *microcephalus* is a member of the bower-building ‘*Copadichromis/Nyassachromis/Mchenga*’ species complex, which is widely dispersed throughout the lake and contains over sixty species (Konings, 2007), many of which have localised distributions. Within the complex, closely related species often overlap considerably in anatomical characters and nuptial colour patterns, but sympatric populations make species-specific bowers. Females of the species are dull grey coloured but breeding males have metallic blue heads and flanks, and yellow and black on the mid body and ends of the fins (Fig. 1).

The study was conducted using SCUBA off the shore of Thumbi West Island in Lake Malawi National Park (14°01′22 S, 34°49′24 E) in the southern arm of Lake Malawi (Figs. 1A and 1B). Our study population of *N*. cf. *microcephalus* occupies a shallow water lek situated approximately 30 m from shore in water 5–10 m deep. In the years we have studied this lek (2005–2009) it has been occupied almost exclusively by *Nyassachromis* cf. *microcephalus*, with a small and annually varying number of bowers occupied by *Mchenga* sp. (maximum 5%). Before the beginning of the study, we counted all the *N*. cf. *microcephalus* bowers and mapped them on the lek by recording the distance (*D*) and radial coordinates (*A*) of each bower from an approximate central position in the lek. The location where bower density was the highest was defined as the centre of the lek. The radial coordinate (in degrees) and the distance from the centre (in cm) were then used to estimate the *x* and *y* coordinates of each bower on the lek (*x* = cos(*A*∗*Pi*/180)∗*D*; *y* = sin(*A*∗*Pi*/180)∗*D*) (Young et al., 2009). The *x* and *y* coordinates of bower position on the lek were reduced to one Principal Component (spatial PC1), which was then used in all further analyses. Bowers further from the centre had lower PC1 values.

**Figure 2:**
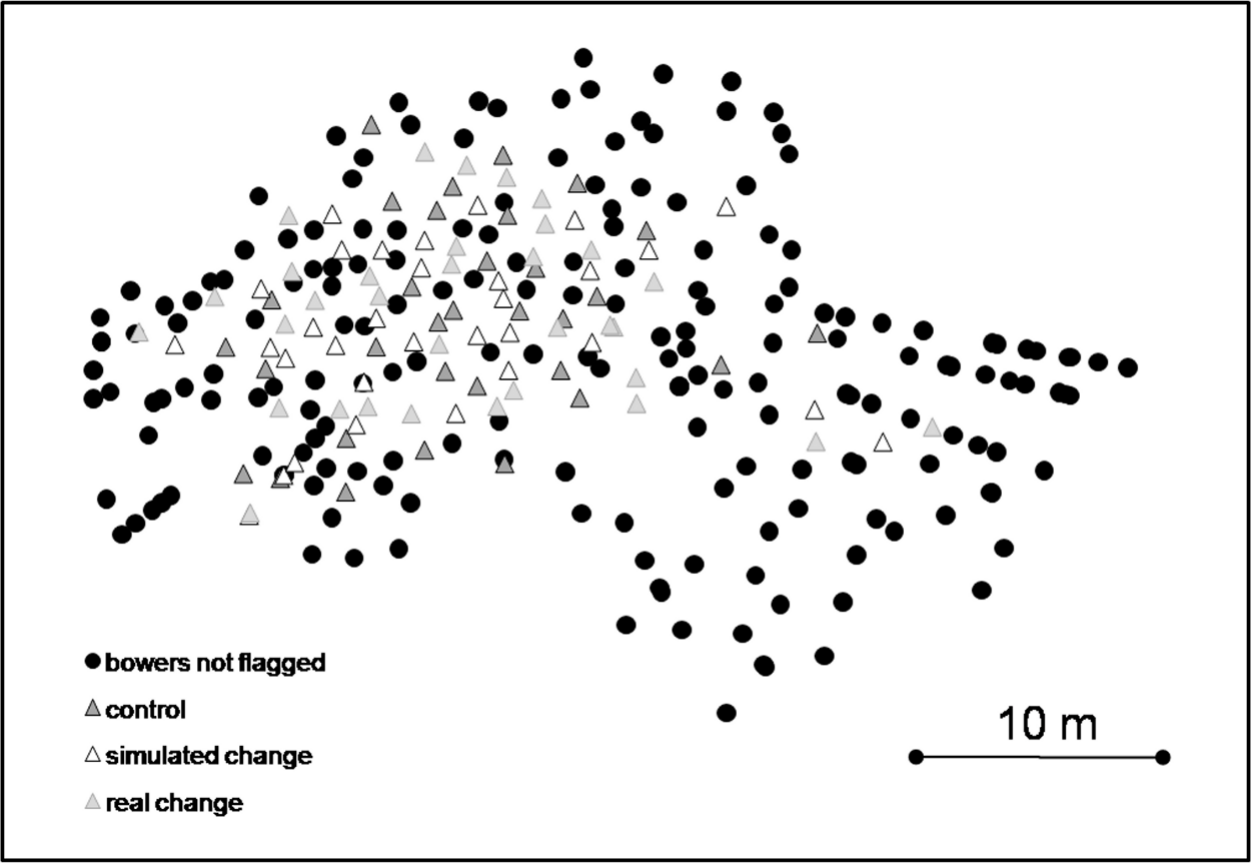
Map of lek with locations of bowers assigned to each treatment and non-flagged bowers.

The whole lek consisted of 297 *Nyassachromis* cf. *microcephalus* bowers (Fig. 2). We randomly selected 99 of these bowers for observations and individually marked them with small numbered flags (Fig. 2).

### Experimental design

We divided the 99 bowers into 3 treatments: “manipulation” (*n* = 33), “simulated manipulation” (*n* = 33) and “control” (*n* = 33). “Manipulation” treatment involved digging a deeper and larger crater and making the bower shape more symmetrical (Fig. 1D), similar to the “volcano” bower shapes built by a number of other species (Kidd, Kidd & Kocher, 2006). We ensured that bower height remained the same through the manipulation, as changes in bower height may affect fish behaviour (Martin & Genner, 2009). “Simulated manipulation” controlled for the effect of disturbance by disturbing the bower but rebuilding it to the original shape. “Control” bowers were not disturbed or manipulated. In all treatments, we recorded male and female behaviour counts before and after manipulations of bower shape were performed.

All data were collected by two observers after first ensuring repeatability of tallies between observers. Observations were done between the 29th of July and the 12th of August 2009, between 8.00 and 11.00 in the morning. It was impossible to observe all 99 bowers on the same day, therefore bowers were divided into six groups, five of which had 18 bowers (Group 1–5), and the sixth group with nine bowers. Group 1 was observed and manipulated on days 1 and 2, group 2 on days 3 and 4, and so on. On day one, each observer recorded the behaviour of nine males for eight minutes per bower and measured their bowers. On the consecutive day the same bowers were divided into the treatments, and “manipulation” and “simulated manipulation” bowers were subjected to treatment and measured. One hour later behavioural observation and bower measurement was carried out again on all treatments (Table 1).

**Table 1:** Experimental design used in the field experiment. Pre-manipulation observations were done one day before the manipulations and the second observations were done.

Treatment^*^	Observation time before manipulation (min per bower)	Time between manipulation and observation (min)	Observation time after manipulation (min per bower)	*N*
Control	8	-	8	33
Simulated manipulation	8	60	8	33
Manipulation	8	60	8	33

**Notes.**

* Pre-manipulation observations were done one day before the manipulations and the second observations were done.

Three male aggressive behaviours were tallied during the eight minute observations: conspecific male aggression (from here on “CSM”), aggression towards conspecific females (from here on “FA”) and all heterospecific aggression (from here on “HET”). We considered an aggression, any aggressive interaction that involved the focal male, whether he initiated the interaction or responded to an intruder, and independent of the response to the intruder. Heterospecific species were typically *Mylochromis* and *Protomelas* spp. foraging in and on the substrate where the lek was situated. We counted CSM as the number of times a conspecific male invaded the territory of the focal male and the focal male defended himself (Video S2). HET and FA consisted of the number of times a heterospecific male or a female respectively invaded the territory of the focal male, such invasion always resulted in the focal male defending himself. We also counted the number of times a male courted passing females (from here on “COURTS”) (Video S3) and the number of building events (“BUILD”) (Video S4), which consisted of the number of times a male picked up sand with its mouth and dropped it on the bower; this was the vast majority of bower altering behaviours observed.

In order to see if changes in bower shape could affect female response to male courtship, we also recorded the response of females that males courted. We tallied three different female behaviours: female follows male into bower after courtship (“VISITS”), female and male circle (“CIRCLE”), female spawns (“SPAWNS”) (Young et al., 2010).

For each bower for which these behaviours were recorded, 12 measurements of bower shape were also taken (Fig. 1D): maximum and minimum bower height (A1, A2), maximum and minimum bower base width (B1, B2), top platform outer (C1) and inner (D1) diameter along the maximum–minimum line and its 90° measurements (C2, D2), maximum and minimum slope of bower platform (E1, E2), bower slope at maximum and minimum height (F1, F2).

### Bower shape analysis

In order to reduce the number of bower measurements to fewer variables, we conducted a Linear Discriminant Analysis on the 12 bower measurements. The three treatments were used as a grouping variable, therefore two Discriminant Functions (DF1, DF2) vectors were obtained. The Discriminant Function scores were then used in all further analysis. In order to confirm that bower shape had been significantly altered in the “manipulation” treatment and not in the other treatments we performed a MANOVA comparing the Discriminant Functions at the time of each measurement for each treatment separately.

### Analysis of male behaviour

All analyses were performed using the statistical software R 2.14.2 (http://www.r-project.org/). We used generalised linear mixed effects models with Poisson error distributions to statistically assess the effect of bower shape upon the five male behaviours. Explanatory variables in the models were treatment (control, simulated manipulation and manipulation), time (before and after) and their interaction terms. To account for the non-independence of behaviours recorded for the same individual at the two different times we included individual ID as a random effect.

A previous study on this lek found that bower distance from the centre of the lek is negatively correlated with aggression (Young et al., 2009). So in order to control for the effect of location of each bower on the lek spatial PC1 was included in the model as a covariate (Young et al., 2009; Young et al., 2010). Note that throughout the ‘Results’ section we only report significant interaction terms.

### Analysis of female response to male courtship

We assessed females’ response to male courtship using mixed effects models similar to the ones used to analyse male behaviours. However, males tend to court any females that pass by their bower and female behaviours are strongly correlated with male courtship behaviour (Genner et al., 2008; Young et al., 2009). In order to control for the effect of male courtship initiation we included male courting as a covariate in the model and two female behaviour categories (VISITS and CIRCLE) as dependent variables. As spawning was only observed once, this behaviour category was excluded from any analysis.

## Results and Discussion

In a Lake Malawi bower-building cichlid lek, we analysed the behaviour of males from 99 bowers and the behaviour of the females that visited these bowers, for a total of 26.5 h.

### Verification of experimental design

We first confirmed that bowers assigned to different treatment groups were equally distributed throughout the lek (ANOVA, *F*_2_ = 0.336, *P* = 0.716), that the two Discriminant Functions (DFs) of bower shape did not differ significantly (MANOVA: *F*_4_ = 0.599, *P* = 0.664) and that the eight behaviour observation categories did not differ significantly (MANOVA: *F*_14_ = 0.937, *p* = 0.520). The 12 bower shape measurements were reduced to two DFs, the first explained 89% of the variance among bowers and base width (B), top platform outer diameter (C), top platform inner diameter (D) and top platform slope (E) had the highest coefficients on this function (Table 2). Top platform outer diameter (C) and top platform inner diameter (D) had the highest coefficients on DF2, which accounted for 11% of the total variance. In the “control” treatment and “simulated manipulation” treatment, the two DFs of bower shape were not significantly different at the time of the first and second observation (i.e., after treatment) (“control” MANOVA: *F*_4_ = 1.081, *P* = 0.346, “simulated manipulation” MANOVA: *F*_4_ = 0.817, *P* = 0.516). We confirmed that the “manipulation” treatment resulted in significant differences in bower shape between the three different times at which those bowers were measured (MANOVA: *F*_4_ = 25.159, *P* < 0.001). Post-hoc tests revealed that both DF1 and DF2 were significantly lower at time of the first observation than immediately after changing the form of the bowers (DF 1: mean difference = −0.636, *P* = 0.047; DF2: mean difference = −0.635, *P* = 0.047) and at the time of the second observation (1 h after the change was made) (DF1: mean difference = 1.232, *P* < 0.001; DF2: mean difference = 1.399, *P* < 0.001). In the “manipulation” treatment, no significant differences were observed between the bower shape immediately after manipulation and one hour later (DF1: mean difference = 0.001, *P* = 1; DF2: mean difference = 0.167, *P* = 0.665).

**Table 2:** Standardized canonical discriminant function. Coefficients of 12 standard measurements on bower shape and the percent variance explained by each function. Traits with highest coefficients are in bold.

Measurement	DF1	DF2
A1 (maximum bower height)	−0.29	−0.08
A2 (minimum bower height)	0.23	0.44
B1 (maximum bower base)	**−0.76**	0.15
B2 (minimum bower base)	0.25	−0.10
C1 (top platform outer diameter)	0.37	0.00
C2 (line perpendicular to C1)	**−0.75**	**−1.05**
D1 (top platform inner diameter)	**−0.55**	−0.09
D2 (line perpendicular to D1)	**0.64**	**1.65**
E1 (maximum slope of bower platform)	**0.59**	0.41
E2 (minimum slope of bower platform)	0.45	0.45
F1 (bower slope at maximum height)	0.40	−0.04
F2 (bower slope at minimum height)	0.25	−0.46
% variance explained	89	11

### Aggressive interactions with conspecific males

Among all treatments, a total of 26.5 h of male behaviour observation documented 3973 counts of building (BUILD), 600 counts of conspecific male aggression (CSM), 1230 counts of heterospecific aggression (HET), 268 counts of aggression towards females (FA) and 475 courts of females (COURTS), (see Table 3 for detailed numbers per treatment and time).

**Table 3:** Numbers of male and female behaviours and their 95% confidence intervals counted by treatment, before and after each treatment, and totals.

	Control		Manipulation		Simulated manipulation	
	*N*	95%CI	*N*	95%CI	*N*	95%CI
**Bower building (BUILD)**						
Before	577	3.732	663	3.670	652	3.231
After	787	4.184	678	3.469	616	4.327
Total	1364	2.858	1341	2.517	1268	2.694
**Conspecific Male Aggression (CSM)**						
Before	113	1.31	112	0.665	89	0.758
After	131	1.222	73	0.899	82	0.806
Total	244	0.891	185	0.572	171	0.553
**Female Aggression (FA)**						
Before	32	0.434	42	0.591	40	0.834
After	38	0.543	44	0.659	72	1.156
Total	70	0.344	86	0.440	112	0.721
**Heterospecific Aggression (HET)**						
Before	179	1.396	143	0.943	183	1.456
After	208	1.549	204	2.324	313	3.059
Total	387	1.041	347	1.263	496	1.757
**Male courts female (COURT)**						
Before	82	0.739	80	0.735	59	0.698
After	73	0.705	86	0.688	95	0.712
Total	155	0.508	166	0.500	154	0.516
**Females follows male into bower (VISIT)**						
Before	11	0.251	13	0.234	5	0.12
After	10	0.24	8	0.167	14	0.222
Total	21	0.172	21	0.144	19	0.131
**Female and Male circle (CIRCLE)**						
Before	0	-	3	0.090	1	0.057
After	4	0.191	1	0.058	8	0.188
Total	4	0.094	4	0.056	9	0.101
**Female Spawns (SPAWN)**						
Before	0	-	0	-	0	-
After	1	-	0	-	0	-
Total	1	-	0	-	0	-

**Figure 3:**
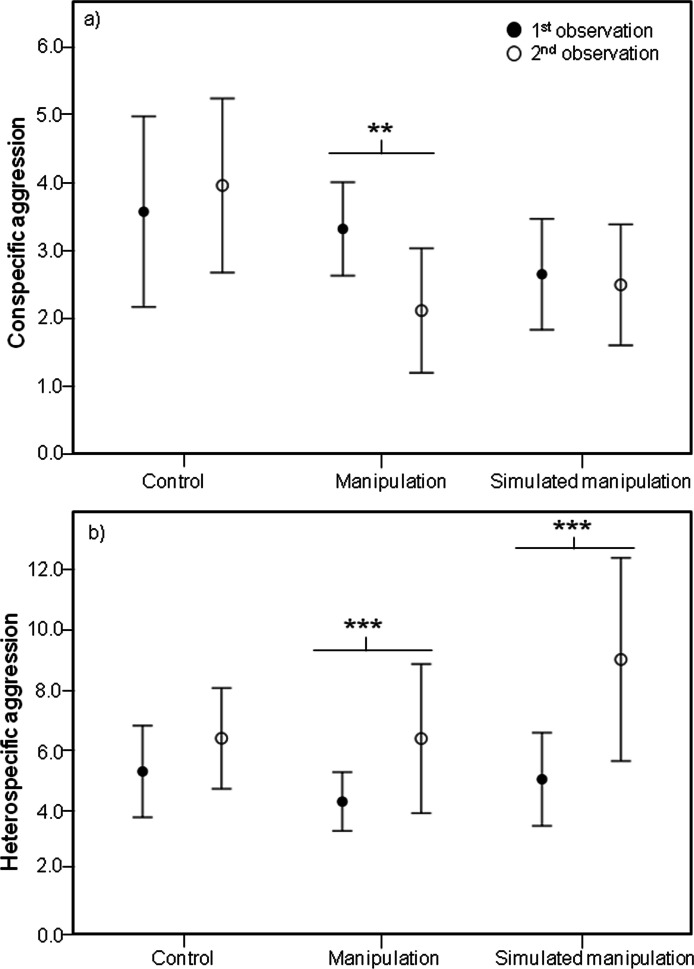
Mean conspecific and heterospecific male aggression. Mean and 95% confidence intervals of (A) Conspecific Male Aggression (CSM) and (B) Heterospecific Male Aggression (HET) during the first (black circles) and second (white circles) observations for treatments control, manipulation and simulated manipulation. Significant differences between observation times represented by * (*p* < 0.05), ** (*p* < 0.01), *** (*p* < 0.001).

Analysis of these five male behaviours showed significant effects of the interaction between time and treatment on several male behaviours, including a significant effect on conspecific male agression (Chi-square = 8.037, df = 2, *P* = 0.018). Specifically, during the second observations, males in the “manipulation” treatment were involved in less aggressive interactions with conspecific males than during the first observations (*Z*-value = −2.973, *P* = 0.002) (Fig. 3A).

The decrease in male aggressive interactions with conspecific males cannot be explained by males focusing more on rebuilding their bowers. Although the interaction between time and treatment had a significant effect on building (Chi-square = 24.317, df = 2, *P* < 0.001), surprisingly only within the control treatment did males build more during the second observations than during the first ones (*Z*-value = 5.108, *P* < 0.001).

Changing the bower shape results in a decrease in aggressive interactions with other males of the same species. There are two possible explanations for this. First, bower modification may have lead to a change in a male behaviour we did not measure which indirectly affected male aggression. We could not differentiate between receiving and initiating conspecific male aggression in this study, and it could be that bower owners with altered bowers initiate less aggression. Bower shape may have had some cognitive or physiological effect on the owner, and therefore influenced his behaviour and other fitness related traits. This would imply that these fish can assess the shape of their extended phenotype and change their behaviour accordingly, in a similar way to satin bowerbirds (Bravery & Goldizen, 2007), an idea that could be tested under laboratory conditions.

The alternative explanation is that males with an altered bower shape may experience an advantage by receiving less aggression from other males as a result of this difference in shape. This idea is in line with theoretical work that has suggested negative frequency-dependent (natural or sexual) selection as a likely mechanism for the maintenance of polymorphisms (Mikami, Kohda & Kawata, 2004; Seehausen & Schluter, 2004; Van Doorn, Dieckmann & Weissing, 2004). As the new phenotype becomes more common this advantage should then decrease. Here, the frequency of bower shape may then oscillate but negative frequency-dependent intrasexual selection generated by male-male-aggression would allow for the emergence of a balanced polymorphism in bower shape. Empirical evidence for the idea that negative-frequency dependent selection can allow for the emergence and maintenance of polymorphisms is so far scarce (but see Hori, 1993; Olendorf et al., 2006; Dijkstra, Seehausen & Groothuis, 2008; Dijkstra, van der Zee & Groothuis, 2008). Our study seems to support this theory.

### Heterospecific aggression after manipulation

It is important that reduced conspecific aggression towards males with differently shaped bowers is not outweighed by increased aggression from heterospecific fish, otherwise any potential selective advantage is negated. We did find a significant effect of the interaction between time and treatment on heterospecific aggression (Chi-square = 7.419, df = 2, *P* = 0.025). During the second observations, males in both the “manipulation” and “simulated manipulation” treatments were involved in more aggressive interactions with heterospecific fish (manipulation: *Z*-value = 3.678, *P* < 0.001; simulated manipulation: *Z*-value = 5.881, *P* < 0.001) (Fig. 3B) than during the first observations. This is likely to be an effect of substrate disturbance releasing food particles and attracting foraging species. This is supported by the increase in heterospecific aggression in both manipulation treatments (Fig. 3B). The reduction in conspecific male aggression we recorded is unlikely to be a result of males shifting their time allocation budget to deal with the increase in heterospecific aggression, because conspecific male aggression did not decrease in both treatments where heterspecific aggression increased (Fig. 3A).

**Figure 4:**
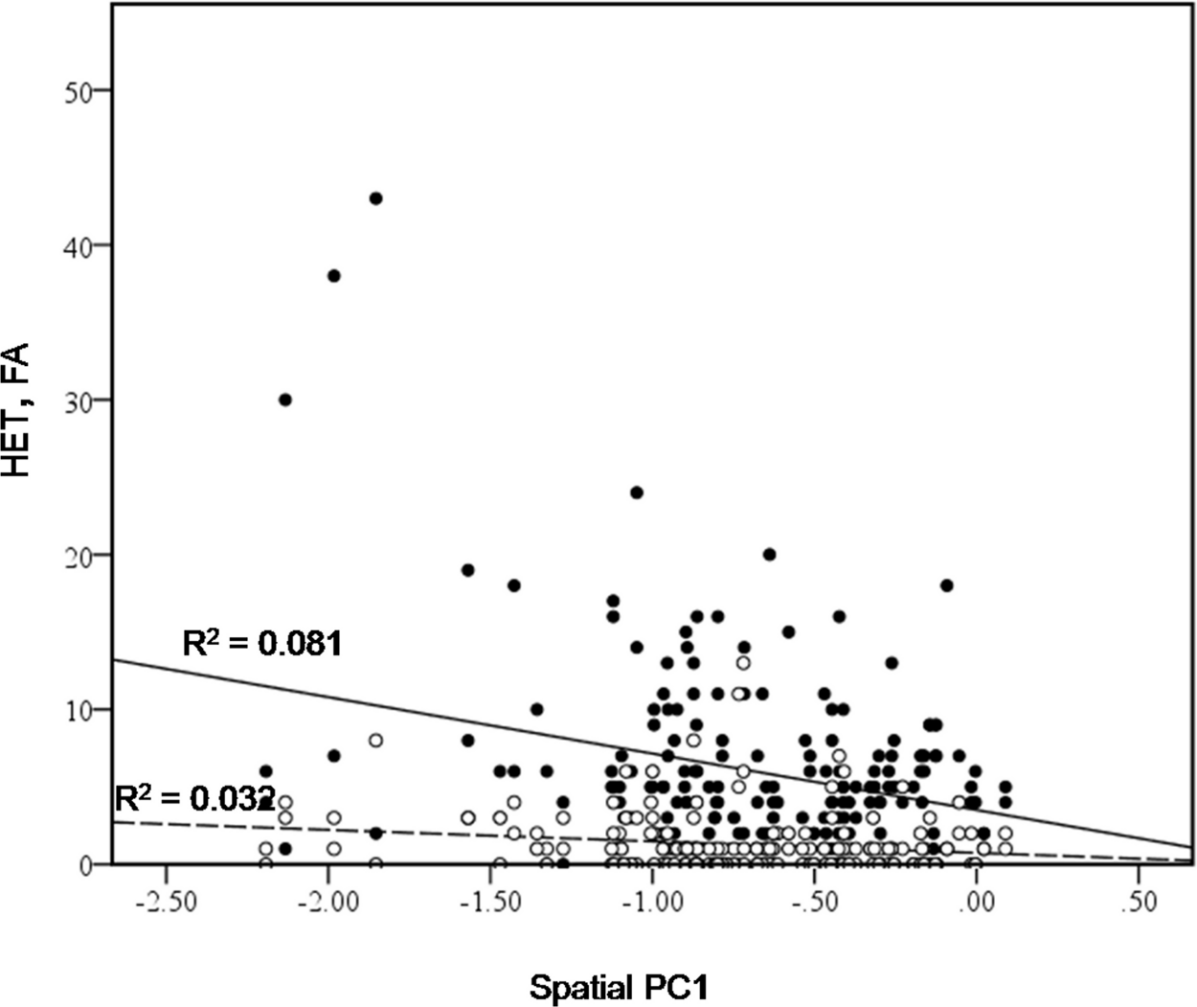
Bivariate plot of counts of heterospecific male aggression (HET, black dots) and aggression towards females (FA, white dots) versus spatial PC1.

We also found that bower location played a role; spatial PC1 was found to have a significant effect on heterospecific aggression (HET) (Chi-square = 9.5469, d.f. = 1, *P* = 0.002), and on aggression towards females (FA) (Chi square = 5.538, d.f. = 1, *P* = 0.0186) (Fig. 4). Males with bowers further from the centre of the lek appear to be involved in more aggressive interactions with other species. This could provide an indirect advantage to males holding a bower with a different form. If males with different bowers receive less aggression from conspecific males, they might be more able to benefit from holding a central position on the lek. This would offer protection from other species. In addition to this indirect advantage, location on the lek appears to provide a direct advantage, as previous studies found that males in the centre of the lek received more visits from females (Kellogg, Stauffer & Mckaye, 2000).

### Female (direct) mate preference

If negative-frequency-dependent intrasexual selection due to male-male aggression is a possible mechanism for maintaining diversity until reproductive isolation can emerge, any advantage conferred by reduced aggression towards males with rare phenotypes must not be outweighed by direct mate choice against those phenotypes, otherwise there is no net benefit to possessing novelty. The use of multiple signals to perform different functions is one way to avoid this potential trade-off (e.g., in red-collared widowbirds, Andersson et al., 2002). However, here, where bowers are a signal to both males and females, we found no evidence for a disadvantage in having a different bower in terms of female preference. One idea which remains to be tested is the possibility that polymorphism in female preference could also emerge and be maintained by frequency-dependent disruptive selection (Van Doorn, Dieckmann & Weissing, 2004). Additionally, the combined interactions of heterospecific aggression allowing novel bower owners to occupy central territories, and arising female preference for those territories would also facilitate the invasion of novel phenotypes (Dijkstra, Seehausen & Groothuis, 2008; Dijkstra, van der Zee & Groothuis, 2008).

After bowers were manipulated, males courted females and females visited their bower as much as before they were manipulated. Courting behaviour was slightly affected by the interaction of time and treatment (Chi-square = 7.357, d.f. = 2, *P* = 0.025), but only within the treatment “simulated manipulation” was this effect significant: males courted more females during the second observations than the first ones (*Z*-value = 2.876, *P* = 0.004). Females were observed following males into their bower (VISIT) 61 times, 17 circled (CIRCLE) and one female spawned (SPAWN) (see Table 3 for detailed numbers per treatment and time). When individual female behaviours were analysed separately male courting appeared to have an effect on numbers of females following males into the bowers (Chi-square = 15.535, d.f. = 1, *P* < 0.001). No other significant effects were observed on female behaviours.

This is consistent with a study on another bower building cichlid *Hemitilapia oxyrhynchus*, which found that the number of females’ visits to a bower were not affected by changes to its shape and that only the frequency of male courting explained female preference (Genner et al., 2008). Females will very often leave the bower that they are visiting at any point of the courting sequence. Based on our data we estimated that only 13% of the females that were courted followed the male into his bower, 36% of those circled with them, and only 6% of those laid eggs with them. So, in total only 0.2% of courts by a male were successful. Female mate choice appears to be a complex process made up of multiple stages where females make sequential stage-specific decisions based on the assessment of male display traits such as courting and bower shape and size (Young et al., 2010; Schaedelin & Taborsky, 2010; Candolin, 2003). Such processes have also been documented in other taxa, e.g., satin bowerbirds (Coleman, Patricelli & Borgia, 2004). The ultimate test of reproductive advantage can only be measured in terms of number of offspring fathered, and this remains to be quantified. Nonetheless our results suggest that bower shape polymorphism could emerge and establish itself even if female preference has not yet arisen. It is known that the species we studied here has polymorphic bowers: some males build similar asymmetrically shaped bowers but on rocks, instead of the far more common asymmetric sand volcanoes that we observed (Konings, 2007; Martin & Genner, 2009; Martin, 2010). Future studies could address potential reproductive isolation between adjacent rocky and sand populations, which could be due to differences in bower shape. Indeed, since male bowers are used in competitor recognition, agonistic character displacement (Grether et al., 2009) could be responsible for the further divergence of populations/incipient species even without a corresponding link to female preference.

## Conclusions

We found that on a lek of bower building cichlid fish from Lake Malawi, males provided with a rare and altered extended phenotype (bower shape) were involved in less aggressive interactions with other males than males holding bowers of the normal extended phenotype. This could confer a selective advantage to novel bower holding males when trying to establish themselves on the lek due to reduced aggression from conspecific males. Additionally, we found no evidence for a disadvantage in having a different bower shape in terms of female preference. The advantage of novelty is apparently not overridden by a disadvantage through female preference against the novelty, so that a new bower shape could become established in the population. Even if female preference has not yet emerged, negative frequency-dependent intrasexual selection is a likely mechanism for the establishment and coexistence of a polymorphism in bower shape.

This is a mechanism that could explain the diversity of bower shape found at several locations across Lake Malawi, where several species coexist on the same lek (Konings, 2007). Further supporting our conclusions, a large scale meta-analysis of 52 species on 47 habitat islands in Lake Victoria reported that territories of males of the same colour are negatively associated on the spawning site, and that the distribution of closely related species over habitat islands is determined by nuptial coloration (Seehausen & Schluter, 2004). The authors suggest that negative-frequency-dependent intrasexual selection due to male-male aggression may be a more common mechanism than previously thought and an important force for the diversification of these organisms.

## Supplemental Information

10.7717/peerj.209/supp-1 Video S1 Lek overview Overview of the bower-building cichlid Nyassachromis cf. microcephalus lek near Thumbi West Island, in southern Lake Malawi.

10.7717/peerj.209/supp-2 Video S2 Male defending territory Conspecific Male Aggression behaviour: the focal male adopts an aggressive posture by opening his fins and showing his side to the conspecific male that invaded the area of his bower and then chases him.

10.7717/peerj.209/supp-3 Video S3 Male courting female Courting behaviour: Male makes courting movements to passing by female. In this specific situation female ignores male.

10.7717/peerj.209/supp-4 Video S4 Bower building behaviour Building behaviour: male picks up sand with his mouth from the vicinity of his bower and drops the sand on top of the bower.

## Additional Information and Declarations

### Field Study Permissions

The following information was supplied relating to ethical approvals (i.e., approving body and any reference numbers): Malawi Fisheries Institute and the Malawi National Park gave permission for this work in person.
